# The local wavenumber model for computation of turbulent mixing

**DOI:** 10.1098/rsta.2021.0076

**Published:** 2022-03-21

**Authors:** Susan Kurien, Nairita Pal

**Affiliations:** Fluid Dynamics and Solid Mechanics (T–3), Theoretical Division, Los Alamos National Laboratory, Los Alamos, NM 87545, USA

**Keywords:** turbulence, turbulence closure models, spectral closure models

## Abstract

We present an overview of the current status in the development of a two-point spectral closure model for turbulent flows, known as the local wavenumber (LWN) model. The model is envisioned as a practical option for applications requiring multi-physics simulations in which statistical hydrodynamics quantities such as Reynolds stresses, turbulent kinetic energy, and measures of mixing such as density-correlations and mix-width evolution, need to be captured with relatively high fidelity. In this review, we present the capabilities of the LWN model since it was first formulated in the early 1990s, for computations of increasing levels of complexity ranging from homogeneous isotropic turbulence, inhomogeneous and anisotropic single-fluid turbulence, to two-species mixing driven by buoyancy forces. The review concludes with a discussion of some of the more theoretical considerations that remain in the development of this model.

This article is part of the theme issue ‘Scaling the turbulence edifice (part 2)’.

## 1. Background and motivation for this survey

Since its introduction in [1], a considerable amount of effort has been invested in the development and testing of the so-called local wavenumber (LWN) model—a practical computational model for turbulence that employs suitable closures for a two-point statistical description of turbulence. There is now a substantial body of relatively recent work that we wish to survey in this paper.

While the Navier–Stokes equations are the accepted model for turbulence, they are notoriously expensive to compute for even simple flows and with today’s relatively powerful computers. The computational challenge is compounded in complex multi-physics applications in which turbulence is often just one component. Statistical models sacrifice the details of the flow at every point in space and time, in favour of computing the dynamics of correlation functions of velocity fluctuations using a Reynolds (mean and fluctuations) or similar decomposition. In the process of formulating the statistical correlation equations starting from the Navier–Stokes equation, the well-known ‘closure’ problem arises for which at each order of expression of the statistics, the next higher-order appears due to the nonlinearity of the Navier–Stokes equations. To then ‘close’ the equations, one must typically postulate physically reasonable relationships between the unknown higher-order statistics and the known (computed) lower-order ones. Typically, the closure is performed at the level of second-order correlation for which the triple-correlations need to be modelled. This approach for computing turbulence sacrifices the details of the flow at every point in favour of relatively inexpensively obtaining a description of low-order statistical quantities such as Reynolds stress and density correlations, which is often sufficient for practical applications.

Statistical models for turbulence with point-wise correlations as variables are known as *single-point* models and are widely used in many practical and industrial applications. Examples include those in the Reynolds-averaged Navier–Stokes family of models such as the k-ϵ [2], and k-ω models [3,4], where k is the turbulent kinetic energy, ϵ is the energy dissipation rate and ω is the specific dissipation rate. A similar single-point phenomenological model was introduced by [5] and forms the basis of turbulent mixing models (known as the BHR models) in multi-physics codes widely in use for industrial and research applications [6,7]. The LWN model in [1] was primarily proposed as an improvement over single-point models; specifically, the authors were interested in overcoming the limitation of having to prescribe *a priori* an equation for the energy dissipation rate and, effectively, an assumption of statistical (spectral) equilibrium or near-equilibrium. Single-point models have intrinsic difficulty with predicting phenomena such as strong transients (e.g. in the transition to turbulence) or density variations [8–11]. This is because they do not have information on the multiple scales generated by nonlinearities that are intrinsic to turbulence. Thus, certain flow properties may be better described using *two-point* statistical models, which by definition have variables depending on two points in space, and hence on the scale defined by their separation distance.

A recent review by Zhou [12] comprehensively summarizes the history of spectral models. The study of constant density (single fluid) homogeneous isotropic turbulence via spectral models could be said to have begun with Kraichnan’s direct interaction approximation (DIA) [13] expressed as integro-differential equations for the response function and correlation of velocity fluctuations [13–15]. Although DIA proved to be a breakthrough in understanding the energy cascade and scale interaction in turbulence [16–18], it is expensive due to the need to compute potentially long time-history integrals. Several efforts were made to reduce the complexity of DIA, such as the random coupling model [19] and the Edwards–Herrings approach [20–22]. DIA also has the drawback that it approximates the nonlinear forcing of velocity as Gaussian and overestimates the role of large eddies [23,24]. Kraichnan himself first recognized and attempted to solve this problem using Lagrangian methods to predict the Kolmogorov spectral scaling exponent correctly with the Lagrangian history DIA [24–26], which was followed by complementary attempts by Herring & Kraichnan [27] and Kaneda with Lagrangian renormalization approximation [28]. Statistical closure theories such as the test field model [29–32], and the Eddy damped quasi normal Markovian (EDQNM) model [16,32–39] were developed as attempts towards more computable models requiring only single-time or Markovian statistical functions to be computed. Some theoretical perspectives and a guide for further reading on the topic of closures and their history may be found in the book by Frisch [40].

EDQNM permitted further developments for homogeneous flows in [41,42]. The modelling of anisotropic contributions in homogeneous turbulence has also been amenable to the EDQNM framework [43–46] with more recent extensions to strongly anisotropic, homogeneous flow, with unstably stratified homogeneous turbulence (USHT) [47], and shear-driven and buoyancy-driven turbulent flows [45]. EDQNM models of buoyancy-driven Boussinesq flows have been studied in [48–51] for the USHT system.

EDQNM includes non-local interactions in the wavenumber space in the closure of the nonlinear terms while LWN is strictly local. Indeed EDQNM, due to it is ability to capture non-local interactions to leading order, has been successfully used in more fundamental studies such as non-locality of turbulence cascade and decay [52], models for helicity cascade [53] and limiting processes in the dissipation of turbulence [54,55]. While EDQNM is a more physically and mathematically rigorous framework than LWN, it perhaps lends itself less well to practical calculations, primarily due to the greater expense of computing non-local interactions. Furthermore, extension to the variable-density (non-Boussinesq) two-fluid mixing case, which is extremely important for practical applications, has not yet been achieved using EDQNM.

After introducing and discussing the model equations, our approach will be to review the LWN model for flows of increasing degrees of complexity, ranging from homogeneous and isotropic turbulence, to inhomogeneous and anisotropic single-fluid turbulence, followed by the more complex problem of two-fluid variable-density mixing. While we are aware of a much wider body of work in spectral models, given the scope and size of this article, we are limiting the review to those efforts most closely relevant to our purpose, especially those emerging from our own research collaborations and publications related to LWN within the past 5 to 6 years.

## 2. Introduction to the LWN model equations

Bésnard *et al.* [1] (referred to hereafter as BHRZ) developed a two-point model for describing turbulent flows that are strongly out of statistical equilibrium. Their aim was to develop a model that captured the physical processes necessary for predicting such flows, but that was also as simple as possible so as to be useful for practical calculations. Therefore, instead of using more formal approaches such as renormalized perturbation theory (e.g. [13,56–59]), they used a phenomenological approach in which the nonlinear and non-local processes of the turbulence are represented by simple terms in the equations that reproduce the correct, known qualitative behaviour. The modelling philosophy behind the BHRZ model is similar in spirit to some of the earlier models such as that of Daly & Harlow [60] and that of Launder *et al.* [61], but extrapolates the modelling ideas in those works to two-point space.

The motivation behind the nomenclature ‘Local wavenumber’ model is as follows. BHRZ assumed that each component of the spectral tensor is advected and diffused in k space in a linear fashion. Following Leith [62], the characteristic time scale for this advection and diffusion is taken as $k^{3/2}E{\left(k\right)}^{1/2}$. Thus a simple combination of advection and diffusion, with the assumed time scale, along with further factors of k determined by dimensional consistency, captures a great deal of the qualitative picture of the k-space cascade. This approach is consistent with more recent work that has shown rigorously that the cascade process is largely due to local triadic interactions [63,64]. This assumption of ‘locality’ thus appears in the nomenclature of the LWN model. In this review of BHRZ and subsequent LWN studies, it is seen that for a range of homogeneous isotropic, inhomogeneous, anisotropic, and finally, variable–density turbulence phenomena, LWN predictions are in agreement with existing physical data and with general theoretical expectations.

### (a) Single-fluid turbulence

We here review the original development in [1] for single-fluid inhomogeneous, isotropic, non-stationary flow. Consider a fluid with density ρ and viscosity ν distributed in a three-dimensional physical space, and its dynamics follows the Navier–Stokes equations, and the incompressibility condition. The velocity field u(x,t) and the pressure field p(x,t) are decomposed into their mean and fluctuating parts as follows: $u(x,t)=\bar{u}(x,t)+u^{\prime }(x,t)$, and $p(x,t)=\bar{p}(x,t)+p^{\prime }(x,t)$, where the overbars represent statistical averages over an ensemble of fluid configurations.

For the present purposes, the statistical quantities of interest are the following: the Reynolds stress tensor components $R_{ij}(x_{1},x_{2},t)=\bar{u_{i}^{\prime }(x_{1},t)u_{j}^{\prime }(x_{2},t)}$, the pressure velocity correlation $\Pi_{i}(x_{1},x_{2},t)=\bar{p^{\prime }(x_{1})u_{j}^{\prime }(x_{2})}$ and the two-point, triple correlation

$R_{imn}(x_{1},x_{2},x_{2},t)=\bar{u_{i}^{\prime }(x_{1},t)u_{m}^{\prime }(x_{2},t)u_{n}^{\prime }(x_{2},t)}$, the turbulent energy stress tensor $E_{ij}(x,t)=R_{ij}(x,t)/2$ and the mean–flow gradient tensor U(x,t) with components $U_{ij}=\partial {\bar{u}}_{i}/\partial x_{j}$. Centre and relative coordinates, x and r, respectively, are introduced by the transformations $x=(x_{1}+x_{2})/2$ and $r=x_{1}-x_{2}$. The wavevector (k) space representations are obtained by taking the Fourier transforms of the concerned variables with respect to r as follows: $R_{ij}(x,k,t)=\int \;{\text{e}}^{-\text{i}k\cdot r}R_{ij}(x+\frac{1}{2}r,x-\frac{1}{2}r,t)\;\text{d}r,R_{imn}(x,k,t)=\int \;{\text{e}}^{-\text{i}k\cdot r}R_{imn}(x+\frac{1}{2}r,x-\frac{1}{2}r,t)\;\text{d}r$. The energy and stress-spectra, as functions of wavenumber k=||k||, are obtained as k-shell integrals, proportional to averages over directions in k–space

2.1 $$E_{ij}(x,k)=\frac{1}{2}\int R_{ij}(x,k)\frac{k^{2}\text{d}\Omega_{k}}{4\pi },$$

where $\text{d}\Omega_{k}=\sin\theta \;\text{d}\theta \;\text{d}\phi$ for 0≤θ≤π; 0≤ϕ≤2π. We define $E=\text{trace}[E_{ij}]$ and deviator ${\tilde{E}}_{ij}=(\delta_{ia}\delta_{jb}-(1/3)\delta_{ij}\delta_{ab})E_{ab}$.

Here, we summarize the principal steps and assumptions leading to the LWN equations. First, from the Navier–Stokes equations, obtain the equations for the two-point generalized Reynolds tensor using the familiar techniques of ensemble averaging. Formally, the two-point correlation equations can be expressed as

2.2 $$\frac{\partial }{\partial t}R_{ij}(x_{1},x_{2},t)=\nu (\nabla_{1}^{2}+\nabla_{2}^{2})R_{ij}(x_{1},x_{2},t)+\text{Mean Flow}+\text{Transfer}.$$

The ‘mean-flow’ terms represent the terms coupled to the mean velocity and its derivatives, and account for the exchange of energy and stress between mean flow and turbulence. The ‘transfer’ terms represent the triple correlations of velocities, and do not affect the total energy and stress in the system. They account for cascades in k (spectral) space, i.e. redistribution of energy and stress among different length scales; the exchange of stress among the different tensor components; and the diffusion of turbulence in x (physical) space. The standard transformation from ($x_{1}$, $x_{2}$) to (x, r) coordinates is made and the Fourier transform applied on the vector scale r to obtain (see appendix A of [1] for details) the following:

2.3 $$\begin{array}{rl} & \frac{\partial }{\partial t}R_{ij}(x,k)=\underset{\text{viscous dissipation}}{\underbrace{-2\nu k^{2}R_{ij}(x,k)}}+\underset{\text{viscous diffusion}}{\underbrace{\frac{1}{2}\nu \nabla^{2}R_{ij}(x,k)}}\underset{\text{MF advection}}{\underbrace{-{\bar{u}}_{n}(x)\frac{\partial }{\partial x_{n}}R_{ij}(x,k)}}+\underset{\text{MF coupling}}{\underbrace{\left(\partial {\bar{u}}_{n}/\partial x_{m})k_{n}(\partial /\partial k_{m})R_{ij}(x,k\right)}}\\ & \;\underset{\text{MF coupling}}{\underbrace{-(\partial {\bar{u}}_{i}/\partial x_{m})R_{mj}(x,k)-(\partial {\bar{u}}_{j}/\partial x_{m})R_{im}(x,k)}}\underset{\text{MF-coupled pressure-velocity correlation}}{\underbrace{+2(\partial {\bar{u}}_{n}/\partial x_{m})(k_{n}/k^{2})[k_{i}R_{mj}(x,k)+k_{j}R_{im}(x,k)]}}\\ & \;\underset{\text{return-to-isotropy mechanism from pressure-velocity correlation}}{\underbrace{+i(k_{m}k_{n}/k^{2})[k_{i}R_{mnj}(x,k)-k_{j}R_{mni}(x,-k)]+\frac{1}{2}(k_{m}k_{n}/k^{2})[(\partial /\partial x_{i})R_{mnj}(x,k)+(\partial /\partial x_{j})R_{mni}(x,-k)]}}\\ & \;\underset{\text{turbulent diffusion from pressure-velocity correlation}}{\underbrace{+(k_{m}/k^{2})\left(\partial /\partial x_{n})[k_{i}R_{mnj}(x,k)+k_{j}R_{mnj}(x,-k)\right]-(k_{n}k_{l}/k^{2})(\partial /\partial x_{l})[k_{i}R_{mnj}(x,k)+k_{j}R_{mni}(x,-k)]}}\\ & \;\underset{\text{triple-correlations for turbulent diffusion and transfer}}{\underbrace{\underset{\text{cascading transfer between scales}}{\underbrace{-ik_{n}[R_{inj}(x,k)-R_{jni}(x,-k)]}}\underset{\text{turbulent diffusion}}{\underbrace{-\frac{1}{2}(\partial /\partial x_{n})[R_{inj}(x,k)+R_{jni}(x,-k)]}}}}. \end{array}$$

The terms have been grouped with labels that indicate the physical processes associated with the terms as well as their origins in the Navier–Stokes equation [10]. The first two terms on the RHS of equation (2.3) show viscous dissipation and viscous diffusion as labelled. The second, third and fourth terms represent advection by the mean flow and coupling to mean-flow gradients, representing exchange of stress between the mean-flow and the turbulence. The fifth, sixth and seventh terms arise from pressure–velocity correlations that are re-expressed in terms of the Reynolds stress and triple-correlations using incompressibility and Green’s theorem to solve for pressure; they include additional mean-flow coupling and triple correlation effects, as indicated. The last two terms are the transfer terms. They are triple correlation terms responsible for cascades in k-space, exchange of stress among different tensor components and turbulent diffusion in x-space.

At this stage, the model retains vector dependence on both x and k and is relatively intractable as a practical model. The authors reduce the dimensionality of the problem at this stage by angle-averaging over the sphere in k-space (see equation (2.1)) to obtain an equation for $E_{ij}(k)$.

#### (i) Model forms for each term

In the process of k-shell-averaging, each term is modelled with expressions that are the simplest, dimensionally correct, rotationally invariant terms satisfying the intrinsic properties of the exact terms:

*Mean flow terms.* The k-shell integral of the mean flow term, i.e. the term labelled as ‘MF-coupled pressure-velocity correlation’ in equation (2.3), $MF=(\partial {\bar{u}}_{n}/\partial x_{m})(M_{mj}^{ni}+M_{mi}^{nj})$, where $M_{mj}^{ni}=\frac{1}{2}\int (2k_{n}k_{i}/k^{2})R_{mj}(k,t)(k^{2}d\Omega_{k}/{\left(2\pi \right)}^{3})$, Note the explicit ${\left(2\pi \right)}^{3}$ normalization of the three-dimensional Fourier-transform operation is reproduced here from [1] as a standard Fourier-transform normalization, which the authors of [1] chose to introduce into $E_{ij}$ rather than embed in the Fourier transform itself; however it does not affect what follows in terms of the modelling choices and we reproduce the integral here mainly to emphasize that it is unclosed and requires a modelling choice. Following the procedure similar to that of Launder *et al.* [61] for a one-point model, M is approximated as a linear function of the E tensor. The justification for a linear expression in E is primarily simplicity, and secondarily because M itself linear in R, that is, the non-k–shell-averaged E. The most general such linear form for M, consistent with the symmetries $M_{nj}^{mi}=M_{nj}^{in}=M_{jn}^{mi}$ can first be expressed in terms of five constants multiplying standard tensor combinations. Additional constraints, namely MF is traceless in i,j, to ensure energy balance between mean and turbulent energies; $M_{mj}^{ii}=2E_{mj}$; $M_{mj}^{nj}=0$; approximate incompressibility $k_{j}R_{mj}=0$–reduce the expression to a single constant $c_{B}$ so that finally, after some algebra:

2.4 $$\begin{array}{rl} MF & \;=c_{B}\left(\frac{\partial {\bar{u}}_{i}}{\partial x_{m}}E_{mj}+\frac{\partial {\bar{u}}_{j}}{\partial x_{m}}E_{im}-\frac{2}{3}\delta_{ij}\frac{\partial {\bar{u}}_{n}}{\partial x_{m}}E_{nm}\right)+(8c_{B}-6)\left(\frac{\partial {\bar{u}}_{n}}{\partial x_{i}}E_{nj}+\frac{\partial {\bar{u}}_{n}}{\partial x_{j}}E_{in}-\frac{2}{3}\delta_{ij}\frac{\partial {\bar{u}}_{n}}{\partial x_{m}}E_{nm}\right)\\ & \;\;+\left(-3c_{B}+\frac{11}{5}\right)\left(\frac{\partial {\bar{u}}_{i}}{\partial x_{j}}+\frac{\partial {\bar{u}}_{j}}{\partial x_{i}}\right)E. \end{array}$$

In the fourth term on the RHS of equation (2.3), i.e. the term labelled as ‘MF coupling’, the order of $k_{n}$ and $\partial /\partial k_{m}$ can be reversed to give another mean-flow coupled term $MF^{\prime }=1/2(\partial /\partial k)(k(\partial {\bar{u}}_{n}/\partial x_{m})M_{ji}^{nm})$, which provides for the transport of energy in k-space induced by the mean flow. The form of the M-tensor developed above can be used to obtain finally

2.5 $$\begin{array}{rl} MF^{\prime } & \;=\frac{\partial }{\partial k}[k(\left(\frac{-3c_{B}}{2}+1\right)\left(\frac{\partial {\bar{u}}_{i}}{\partial x_{m}}E_{mj}+\frac{\partial {\bar{u}}_{j}}{\partial x_{m}}E_{im}-\frac{2}{3}\delta_{ij}\frac{\partial {\bar{u}}_{n}}{\partial x_{m}}E_{nm}\right)\\ & \;\;+\left(\frac{-3c_{B}}{2}+1\right)\left(\frac{\partial {\bar{u}}_{n}}{\partial x_{i}}E_{nj}+\frac{\partial {\bar{u}}_{n}}{\partial x_{j}}E_{in}-\frac{2}{3}\delta_{ij}\frac{\partial {\bar{u}}_{n}}{\partial x_{m}}E_{nm}\right)\\ & \;+\left(c_{B}-\frac{7}{10}\right)\left(\frac{\partial {\bar{u}}_{i}}{\partial x_{j}}+\frac{\partial {\bar{u}}_{j}}{\partial x_{i}}\right)E+\left(\frac{7c_{B}}{2}-\frac{8}{3}\right)\delta_{ij}\frac{\partial {\bar{u}}_{n}}{\partial x_{m}}E_{nm})]. \end{array}$$

Note that MF has one fewer term (tensor combination) compared with $MF^{\prime }$ because the latter has one fewer constraint in that it does not need to be traceless in i,j. The details of this modelling approach appear tedious but it is worth reiterating that the underlying physical motivations that drove the development were deliberately the minimal ones needed to achieve a respectable representation of MF terms.

*Return–to–isotropy term.* The term in the third line of equation (2.3) is the return-to-isotropy (RTI) term arising from the pressure-velocity correlation that only redistributes the stress between the different components of $E_{ij}$, and contributes to the decay of the off-diagonal components. This term, denoted by tensor-mixing (TM) is phenomenologically modelled after k-shell averaging as

2.6 $$TM=c_{M}k\sqrt{kE}\left[\frac{1}{3}E\delta_{ij}-E_{ij}\right]=c_{M}k\sqrt{kE}{\tilde{E}}_{ij}=c_{M}\Phi {\tilde{E}}_{ij},$$

where $c_{M}$ is an appropriate dimensionless modelling coefficient and $\Phi =k\sqrt{kE}$ is the turbulence frequency. The purpose of this term is to drive the anisotropic parts ${\tilde{E}}_{ij}$ towards zero. Such a term, as we will discuss in §5, is not capable of transporting the anisotropy in k-space; it merely damps the anisotropy in a given wavenumber at a fixed rate.

*k-space cascade transfer term.* Although the physical transfer of energy in k-space is non-local, restricting the model for phenomenological purposes to local interactions only results in a combined wave-like and diffusion-like approximation following Leith [62]. A model representing the transfer in k space is therefore proposed as

2.7 $$\text{cascade transfer between scales}=-c_{1}\frac{\partial }{\partial k}\left(k^{2}\sqrt{kE}E_{ij}\right)+c_{2}\frac{\partial }{\partial k}\left(k^{3}\sqrt{kE}\frac{\partial E_{ij}}{\partial k}\right),$$

where $c_{1}$ and $c_{2}$ are constants. This term represents a non-local integral cascade with a wave-like part (the $c_{1}$ term) and a diffusive part (the $c_{2}$ term). For $c_{1}>0$, the wave-like cascade of $E_{ij}$ is always forward (i.e. towards higher wavenumbers), and $c_{2}>0$ results in a forward as well as inverse cascade ([65]). For physically reasonable spectra, indeed $c_{2}\geq 0$, as diffusion is needed to provided energy transfer in both directions in k-space.

*Turbulent self-diffusion term.* The last terms to be modelled are the fourth line and the second term in the last line in the RHS of equation (2.3). These are conservative in physical space, and were modelled as turbulent self-diffusion following [60]. A symmetrized spectral analogue is

2.8 $$c_{D}\frac{\partial }{\partial x_{n}}\left(D_{nl}\frac{\partial }{\partial x_{l}}E_{ij}+D_{jl}\frac{\partial }{\partial x_{l}}E_{ni}+D_{il}\frac{\partial }{\partial x_{l}}E_{jn}\right),$$

where $c_{D}$ is an appropriate dimensionless modelling coefficient. The diffusion coefficients $D_{nl}$ would be expressed as $D_{nl}=\int_{0}^{\infty }\sqrt{k/E}(E_{nl}/k^{2})\;\text{d}k$. It was found by the authors of [1] that a further simplification of the turbulent diffusion was largely sufficient for many applications

2.9 $$\text{turbulent diffusion}=c_{D}\frac{\partial }{\partial x_{n}}\upsilon_{T}\frac{\partial E_{ij}}{\partial x_{n}},$$

where $\upsilon_{T}(x)=\int_{0}^{\infty }(\sqrt{kE(x,k)}/k^{2})\;\text{d}k$.

#### (ii) General model equations for energy tensor

The elements of the model described above will now be assembled into a complete set of evolution equations for the k-shell integrated stress tensor in BHRZ describing spectral transport in inhomogeneous constant-density turbulence

2.10 $$\begin{array}{rl} & \frac{\partial E_{ij}}{\partial t}+{\bar{u}}_{n}\frac{\partial E_{ij}}{\partial x_{n}}=V[E_{ij}]+T[E_{ij}]-\left(\frac{\partial {\bar{u}}_{i}}{\partial x_{n}}E_{nj}+\frac{\partial {\bar{u}}_{j}}{\partial x_{n}}E_{in}\right)\\ & \;\;+c_{B}A_{ij}+(8c_{B}-6)B_{ij}+\left(-3c_{B}-\frac{11}{5}\right)C_{ij}\\ & \;\;+\frac{\partial }{\partial k}k\left[\left(\frac{-3c_{B}}{2}+1\right)A_{ij}+\left(\frac{-3c_{B}}{2}+1\right)B_{ij}+\left(\frac{7c_{B}}{2}-\frac{8}{3}\right)\delta_{ij}\frac{\partial {\bar{u}}_{n}}{x_{m}}E_{nm}\right]\\ & \;\;+c_{M}k\sqrt{kE}\left(\frac{1}{3}E\delta ij-E_{ij}\right)], \end{array}$$

where for compactness the following differential operators are defined:

2.11 $$V=-2\nu k^{2}+\frac{1}{2}\nu \nabla^{2};\;T=c_{D}\frac{\partial }{\partial x_{n}}\upsilon_{T}\frac{\partial }{\partial x_{n}}-c_{1}\frac{\partial }{\partial k}k^{2}\sqrt{kE}+c_{2}\frac{\partial }{\partial k}k^{3}\sqrt{kE}\frac{\partial }{\partial k};$$

and the following tensors are defined:

2.12 $$\begin{array}{r} A_{ij}=\frac{\partial {\bar{u}}_{i}}{\partial x_{n}}E_{nj}+\frac{\partial {\bar{u}}_{j}}{\partial x_{n}}E_{in}-\frac{2}{3}\delta_{ij}\frac{\partial {\bar{u}}_{n}}{\partial x_{m}}E_{nm}, \end{array}$$

2.13 $$\begin{array}{r} B_{ij}=\frac{\partial {\bar{u}}_{n}}{\partial x_{i}}E_{nj}+\frac{\partial {\bar{u}}_{n}}{\partial x_{j}}E_{in}-\frac{2}{3}\delta_{ij}\frac{\partial {\bar{u}}_{n}}{\partial x_{m}}E_{nm} \end{array}$$

2.14 $$\begin{array}{r} \;\text{and}\;C_{ij}=\left(\frac{\partial {\bar{u}}_{i}}{\partial x_{j}}+\frac{\partial {\bar{u}}_{j}}{\partial x_{i}}\right)E. \end{array}$$

The set of equations (2.11) has five independent coefficients that arise from the primary modelling approximations $c_{B}$ (mean-flow coupling effects), $c_{M}$ (return to isotropy), $c_{D}$ (turbulent diffusion), $c_{1}$ and $c_{2}$ (spectral transfer and cascade), which can be tuned based on the flow to be computed. As we will discuss, the cascade coefficients are further constrained by the Kolmogorov constant and the inviscid limit of equipartition [66], leaving, in the case of single-fluid turbulence just three free coefficients.

The general set of model equations (2.11) can be reduced to various specific cases depending on the flow to be studied. Below we will show these reduced forms for the main flows that have been investigated using this model.

*LWN equations for statistically homogeneous flow.* In statistically homogeneous flow, the mean flow velocity is restricted to the form ${\bar{u}}_{m}(x)={\bar{u}}_{m}(0)+U_{mn}x_{n}$, where the components $U_{mn}$ of the mean flow gradient tensor U are assumed constant in space. The two-point correlations depend on r, but are no longer functions of x. Coordinate derivatives applied to the correlations can be replaced with derivatives with respect to r. Equations (2.11) in this case reduce to a set with the inhomogeneous spatial advection terms absent, and two unknown coefficients $c_{B}$ and $c_{M}$ (see [67,68] for the reduced set of equations); as discussed [69] it was realized that with $c_{1}$ and $c_{2}$ are separately constrained.

A further simplification may be made for single-fluid homogeneous isotropic decaying turbulence

2.15 $$\frac{\partial E}{\partial t}=-2\nu k^{2}E-c_{1}\frac{\partial }{\partial k}(k^{5/2}E^{3/2})+c_{2}\frac{\partial }{\partial k}\left(k^{7/2}E^{1/2}\frac{\partial E}{\partial k}\right).$$

As discussed above, the theoretical constraints on the cascade eliminate freedom to tune $c_{1}$ and $c_{2}$, making this an effectively parameter-free model. Homogeneous flow application will be reviewed in §3.

*LWN equations for inhomogeneous turbulence and the shear-free mixing layer.* Retaining the inhomogeneous (x-dependent) transfer terms in equation (2.11), we are considering the special case in which there is no mean-shear and the inhomogeneity is created by a gradient in the turbulent kinetic energy. Suppose the inhomogeneity is in the y- or two-direction only, such that x=(0,y,0). In this case, the turbulent diffusion term in LWN or the BHRZ equation becomes $c_{D}\partial_{y}(D_{22}\partial_{y}E_{ij}+D_{j2}\partial_{y}E_{2i}+D_{i2}\partial_{y}E_{j2})$. While the turbulence frequency is nominally defined as $\Phi =k\sqrt{kE}$ (see equation (2.6) in [1]). A modified frequency was suggested in [70] in order to consider the viscous effects on the turbulence time scales, $\Phi (y,k,t)=(1/2)[{\left(\nu^{2}k^{4}+4H\right)}^{1/2}-\nu k^{2}]$, where $H(y,k,t)\equiv c_{H}\int_{0}^{k}q^{2}E(y,q,t)dq.$ and $c_{H}=2/9$. This version was used in the study of a shear-free mixing layer (SFML) in [71] to consider the viscous effects on the frequency.

2.16 $$\begin{array}{rl} \partial_{t}E_{ij}(y,k,t) & \;=\underset{\text{viscous dissipation and diffusion}}{\underbrace{(-2\nu k^{2}+(\nu /2)\partial_{x}^{2})E_{ij}}}+\underset{\text{return-to-isotropy}}{\underbrace{c_{M}\Phi {\tilde{E}}_{ij}}}\\ & \;\;+\underset{x\text{-space transport ( turbulent self-diffusion) }}{\underbrace{c_{D}\partial_{y}(D_{22}\partial_{y}E_{ij}+D_{j2}\partial_{y}E_{2i}+D_{i2}\partial_{y}E_{j2})}}\underset{k\text{-space transport ( cascade)}}{\underbrace{-c_{1}\partial_{k}(k\Phi E_{ij})+c_{2}\partial_{k}(k^{2}\Phi \partial_{k}E_{ij})}}. \end{array}$$

The SFML application is reviewed further in §4.

### (b) Variable-density turbulence

Constructing a model for variable-density turbulence is important for computation in applications in which materials with different densities mix, typically driven by instabilities. Variable-density flows are those in which fluctuations of the density from its mean value are so large as to cause the flow to depart from the more tractable Boussinesq limit. The LWN equations for the variable-density case are obtained in a manner similar to the constant-density turbulence, the primary difference being the method of averaging. Instead of simple means, we consider density-weighted means of the variables, as shown below. Additional statistical variables also need to be evolved as we show below.

In addition to the Reynolds decomposition of velocity and pressure (see §2a), we decompose the density $\rho =\bar{\rho }+\rho^{\prime }$ and the specific volume $1/\rho =\upsilon =\bar{\upsilon }+\upsilon^{\prime }$ where, as before, the overbar denotes the mean, and the primes the fluctuations about the mean. It is useful to work with the mass-weighted Favre averages [72]. The Favre-averaging is a choice made both due to the history of this particular model and because it renders the energy equation in conservative form. The Favre-averaged velocity $\tilde{u}$ is $\tilde{u}=\bar{\rho u}/\bar{\rho }$. Let $u^{\prime \prime }$ denote the fluctuation about this Favre-averaged velocity $\tilde{u}$. Then, $u=\tilde{u}+u^{\prime \prime }$. Applying the standard Reynolds decomposition to $\bar{\rho u}$, $\bar{\rho u}=\bar{\rho }\bar{u}+\bar{\rho^{\prime }u^{\prime }}$. Simplifying further using the definition of $\tilde{u}$, $\bar{\rho }\tilde{u}=\bar{\rho }\bar{u}+\bar{\rho^{\prime }u^{\prime }},\tilde{u}=\bar{u}+\bar{\rho^{\prime }u^{\prime }}/\bar{\rho }$. The velocity associated with the net turbulent mass flux is defined by $a=\bar{\rho^{\prime }u^{\prime }}/\bar{\rho }$; from the definition of $\tilde{u}$, we can then understand a as the velocity of mass flux relative to $\tilde{u}$.

Analogous to the single-fluid case described above, for two arbitrary points $x_{1}$ and $x_{2}$ in space, the two-point mass-weighted Reynolds stress tensor is defined as

2.17 $$R_{ij}(x_{1},x_{2})=\frac{1}{2}\bar{\left[\rho (x_{1})+\rho (x_{2})]u_{i}^{\prime \prime }(x_{1})u_{j}^{\prime \prime }(x_{2}\right)},$$

the velocity associated with the turbulent mass flux is defined as

2.18 $$a_{i}(x_{1},x_{2})=-\bar{u_{i}^{\prime \prime }(x_{1})\rho (x_{1})\upsilon (x_{2})},$$

and the covariance of the density and specific-volume is defined as

2.19 $$b(x_{1},x_{2})=-\bar{\rho^{\prime }(x_{1})\upsilon^{\prime }(x_{2})}.$$

Subscripts i and j indicate Cartesian components, the specific volume is υ(x)=1/ρ(x) and its fluctuations $\upsilon^{\prime }(x)$ are defined with respect to the mean specific volume. In [47], the Boussinesq case does not employ any special density-weighted averaging but analogies may be drawn between their variable and these up to the normalization by density. The b variable corresponds in that case to the two-point correlation of density fluctuation and is therefore analogous to potential energy.

The model is further developed in spectral space, for which we require Fourier-transformed variables. The familiar procedure from §2a is repeated to obtain the dynamic variables as functions of spatial coordinate x and the angle-averaged wavenumber k, $R_{ij}(x,k)$, $a_{i}(x,k)$ and b(x,k). Henceforth, we will use $R_{ij}$, $a_{i}$ and b to denote the spectral quantities at a certain time t, and will drop their respective arguments.

We next describe LWN model equations for two variable-density flows—a homogeneous variable density flow, and the Rayleigh–Taylor instability-driven flow. Both flows do not have mean-flow coupling and therefore the variable density effects are paramount. Following those, and for completeness, we provide a brief discussion of shear-driven Kelvin–Helmholtz instability (KHI) and shock-driven Richtmyer–Meshkov instability (RMI), both of which are more complex and have not yet been examined in detail using this model.

#### (i) Inhomogeneous variable-density turbulence

A canonical example of this type of mixing is the Rayleigh–Taylor instability, which has recently been reviewed comprehensively from the physics and modelling standpoints in [73–76]. In this case, the flow is induced by the relaxation of a statically unstable density stratification, resulting from a heavy fluid (density $\rho_{1})$ resting above a light fluid (density $\rho_{2}$), with gravity g pointing downwards along coordinate y. Thus the system is inhomogeneous with respect to the vertical direction y. The x−z plane is the horizontal plane and is considered statistically homogeneous.

Following Steinkamp *et al.* [65], we write the mass and momentum conservation Navier–Stokes equations for (inviscid) variable-density flows driven by gravity in the y-direction: $\partial \bar{\rho }/\partial t+\partial \bar{\rho }{\tilde{u}}_{y}/\partial y=0$ for the density conservation, and $\partial \bar{\rho }{\tilde{u}}_{y}/\partial t+\partial \rho {\tilde{u}}_{y}{\tilde{u}}_{y}/\partial y=-\partial \bar{p}/\partial y+\bar{\rho }g-(\partial R_{yy}/\partial y)$ for the momentum conservation. If we take the proper convolutions [1,68], we obtain the evolution equations for the Reynolds stress $R_{ij}$, velocity $a_{i}$, and the covariance of the density and specific-volume b. These equations contain triple correlations of the velocity and density fluctuations that represent the turbulence cascade in k space. Based on the diffusion approximation model proposed by Leith [62], these triple correlation terms are modelled as nonlinear advection and diffusion in k-space [1,62,68]. Thus the LWN equations for the general inhomogeneous mixing of two fluids via the Rayleigh–Taylor instability are [65,77]

2.20 $$\begin{array}{rl} \frac{\partial R_{nn}(y,k,t)}{\partial t} & \;=-\frac{\partial R_{nn}{\tilde{u}}_{y}}{\partial y}+\int_{-\infty }^{+\infty }2a_{y}\frac{\partial \bar{p}}{\partial y}(k\exp\left(-2k|y^{\prime }-y|\right))\;\text{d}y^{\prime }\\ & \;\;+\frac{\partial }{\partial k}\left[k\Theta^{-1}\left[-C_{r1}R_{nn}+C_{r2}k\frac{\partial R_{nn}}{\partial k}\right]\right]-2R_{yy}\frac{\partial {\tilde{u}}_{y}}{\partial y}+C_{d}\frac{\partial }{\partial y}\left(\upsilon_{t}\frac{\partial R_{nn}}{\partial y}\right) \end{array}$$

2.21 $$\begin{array}{rl} \frac{\partial R_{yy}(y,k,t)}{\partial t} & \;=-\frac{\partial R_{yy}{\tilde{u}}_{y}}{\partial y}+\int_{-\infty }^{+\infty }2a_{y}\frac{\partial \bar{p}}{\partial y}(k\exp\left(-2k|y^{\prime }-y|\right))\;\text{d}y^{\prime }\\ & \;\;+\frac{\partial }{\partial k}\left[k\Theta^{-1}\left[-C_{r1}R_{yy}+C_{r2}k\frac{\partial R_{yy}}{\partial k}\right]\right]-2R_{yy}\frac{\partial {\tilde{u}}_{y}}{\partial y}\\ & \;\;+C_{d}\frac{\partial }{\partial y}\left(\upsilon_{t}\frac{\partial R_{yy}}{\partial y}\right)+C_{m}\Theta^{-1}\left(\frac{\delta_{ij}}{3}R_{nn}-R_{yy}\right) \end{array}$$

2.22 $$\begin{array}{rl} \frac{\partial a_{y}(y,k,t)}{\partial t} & \;=-{\tilde{u}}_{y}\frac{\partial a_{y}}{\partial y}+\frac{b}{\bar{\rho }}\frac{\partial \bar{p}}{\partial y}-\left[C_{rp1}k^{2}\sqrt{a_{\hat{n}}a_{\hat{n}}}+C_{rp2}\Theta^{-1}\right]a_{y}-\frac{R_{yy}}{{\bar{\rho }}^{2}}\frac{\partial \bar{\rho }}{\partial y}\\ & \;\;+C_{d}\frac{\partial }{\partial y}\left(\upsilon_{t}\frac{\partial a_{y}}{\partial y}\right)+\frac{\partial }{\partial k}\left[k\Theta^{-1}\left[-C_{a1}a_{y}+C_{a2}k\frac{\partial a_{y}}{\partial k}\right]\right] \end{array}$$

2.23 $$\begin{array}{rl} \frac{\partial b(y,k,t)}{\partial t} & \;=\left(\frac{2\bar{\rho }-\rho_{1}-\rho_{2}}{\rho_{1}\rho_{2}}\right)\frac{\partial \bar{\rho }a_{y}}{\partial y}+\frac{\partial }{\partial k}\left[k\Theta^{-1}\left[-C_{b1}b+C_{b2}k\frac{\partial b}{\partial k}\right]\right]+C_{d}\frac{\partial }{\partial y}\left(\upsilon_{t}\frac{\partial b}{\partial y}\right). \end{array}$$

We have here presented, for completeness, the integro-differential form of the non-local pressure-transport term (second term on the RHS of equation (2.21)). For computational efficiency, this term has been successfully modelled by the local approximation $2a_{y}(\partial \bar{p}/\partial y)$ and used to good effect in many studies [65,77,78]. At this juncture, the turbulent diffusion coefficients $C_{d}$ are considered to be equal for all statistical variables, although in principle they may be allowed to vary. Here, $R_{nn}=\text{trace}[R_{ij}]=2\bar{\rho }E$. The turbulence frequency $\Theta^{-1}=\sqrt{\int_{0}^{k}(k^{2}R_{nn}(k,t)/\bar{\rho })\;\text{d}k}$. In the equations (2.20)–(2.23), the dynamical variables $R_{nn}$, $a_{y}$ and b, respectively, are functions of k and time t. The arguments have been dropped on the RHS of the equations for compactness. ν is the viscosity and κ is the mass diffusion term, which are potentially needed for comparisons with experimental flows. The cascade transfer terms for $R_{nn}(k,t)$, $a_{y}(k,t)$ and b(k,t) in equations (2.20)–(2.23) are written in an analogous manner, as an advection and diffusion model, as the Leith’s model introduced previously (equation (2.7)). The drag between the fluids is described in the mass-flux equation (2.23) by the third term on the RHS. Here, $a_{n}$ is the component of a normal to the fluid interface. The $C_{rp1}$ term represents a drag arising between interpenetrating fluids at different scales [68,79,80]. The $C_{rp2}$ term represents conventional drag governed by the turbulence time scale [68]. Dissipation terms proportional to the diffusion coefficient κ and the kinematic viscosity coefficient ν are also introduced. Note that the pressure-gradient couples to b and $a_{y}$ to drive $a_{y}$ and $R_{nn}$, respectively.

*Homogeneous variable-density bouyancy-driven turbulence.* Suppose we have a distribution of blobs of one fluid dispersed in another fluid in a statistically homogeneous manner. The LWN equations for statistically homogeneous variable-density flow then reduce to a set minus the advection term and the turbulent diffusion (see [65,81]). This case is reviewed in §6a.

*Shear-driven and shock-driven turbulence.* A mean-flow coupled spectral model was constructed for the constant density (single fluid) KHI [1]. In that paper, the calculation was studied for qualitative outcomes but not validated against quantitative data, unavailable at the time. Extension to the more general two-fluid case including variable density effects has not been computed by any spectral model to our knowledge. The RMI is an important test case to consider for any turbulence model, and can be considered as the impulsive acceleration limit of RTI. To accurately predict RMI, LWN needs to be extended to compressible flows—an effort yet to be performed in a systematic manner. From a practical standpoint, variable-density model solutions for unresolved (sub-grid) mixing appear to be sufficient in compressible flow simulations. This is the case for single-point models [82] and is likely to be the case for the two-point LWN class of models as well. In both KH and RM variable density flows the evolution equations for the antisymmetric components of the Reynolds stress tensor would be required to capture vorticity and turnover, depending on the fidelity required of the model. While these are a straightforward extension (e.g. [65,77]), the validation studies against benchmark data have yet to be performed. We expect that near future studies will use numerical and experimental data now available [83] to perform the required validation comparisons.

### (c) Numerical implementation

The spectral model calculations of LWN presented in this paper were performed with a code using a second-order MacCormack scheme [84] for time integration. The code uses an exponential grid for the wavenumber $k=k_{s}\exp\{z/z_{s}\}$, where $k_{s}$ and $z_{s}$ are scale factors and assumed to be equal to unity [1]. The variables computed are, in fact $kR_{nn}$, $kR_{ij}$, $ka_{i}$ and kb. This choice of variables results in the cascade terms retaining a conservation form when expressed in terms of z rather than k. Likewise, the values of the integrals of the spectral quantities are easily determined, e.g. $R_{nn}(t)=\int_{0}^{+\infty }R_{nn}(k,t)\;\text{d}k=\int_{-\infty }^{+\infty }R_{nn}(z,t)(k_{s}/z_{s})\exp\{z/z_{s}\}\text{d}z$. Setting $k_{s}$ and $z_{s}$ equal to 1 gives $R_{nn}(t)=\int_{-\infty }^{+\infty }\exp(z)R_{nn}(z,t)\;\text{d}z$, where $\exp(z)R_{nn}(z,t)=kR_{nn}(k,t)$ The explicit MacCormack methodology is nominally second-order accurate in time and space, and uses two steps. Each of the two steps uses single-sided differences for the first-order derivatives, and the sides at which the differences are evaluated are different for the two steps, i.e. left side for the first step and right side for the second. The boundary conditions at k=1 and $k=k_{\text{max}}$ are set to Neumann (zero flux).

## 3. Homogeneous isotropic decaying turbulence

**Figure 1.**
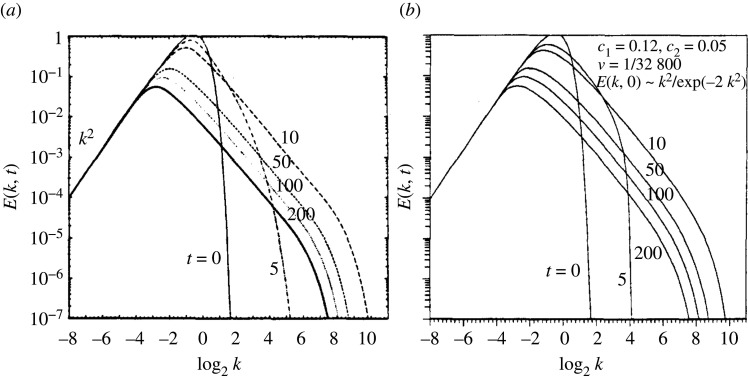
Homogeneous isotropic decaying turbulence spectra computed using (*a*) EDQNM and (*b*) LWN show good agreement. Reproduced from [1].

The original BHRZ model [1] for isotropic homogeneous turbulence produced remarkable agreement with the non-local EDQNM theory for a suitable choice of system coefficients $c_{1}=0.12$ and $c_{2}=0.05$ (see equation (2.16)), thus demonstrating that local theory can match the results of non-local theory (figure 1). The shown shape of the E spectrum becomes independent of time for large t, whereas its normalization and motion on the k axis follow power laws in time. The self-similar spectrum depends on the ratio $c=c_{1}/c_{2}$, and broadly speaking, three regimes are present. For c≥−5/3, there is a $k^{-5/3}$ inertial range, as is expected for three-dimensional turbulence. For c<−3, there is a $k^{-3}$ inertial range, and for −3<c<−5/3, the behaviour is $∼k^{c}$.

Self-similarity of decaying turbulence was examined using LWN in [69], where it was noted that for consistency with the equipartition limit prescribed by Lee [66] and the K41 spectrum coefficient value, $c_{1}/c_{2}=2$ and $c_{1}=0.12;c_{2}=0.06$, very close to the values used in [1] to obtain agreement with EDQNM. Thus in the case of homogeneous, isotropic decaying turbulence, LWN is a parameter-free model.

## 4. Inhomogeneous, anisotropic mixing: the shear-free mixing layer

A non-trivial case of inhomogeneous single-fluid mixing is the shear-free mixing layer, which we discuss next. In this case, the physical-space transport of the turbulent velocity fluctuations becomes the primary mechanism for mixing, which in turn isolates a key component of the model and its assumptions, including an unknown constant $C_{d}$.

In [71], the LWN model was compared with DNS calculations of a SFML [85]. In the SFML, the turbulent kinetic energy propagates from a region of strong turbulence into a region of weak turbulence through a mixing process driven solely by the inhomogeneity. Such flows have also been studied in the laboratory (e.g. [86,87]) and provide useful paradigms for understanding turbulent mixing processes in isolation from more complex mean-flow coupled processes. The main utility of this test case is that it allowed for the testing of the closure used for the physical-space transport of the turbulent velocity fluctuations independently of mean-flow coupled terms, which would add additional modelling complications. For more details on the SFML DNS itself, we invite the reader to review [85] and references there in. The main outcomes from the study in [71] are summarized below. The model equations (2.17) were used in this study.

**Figure 2.**
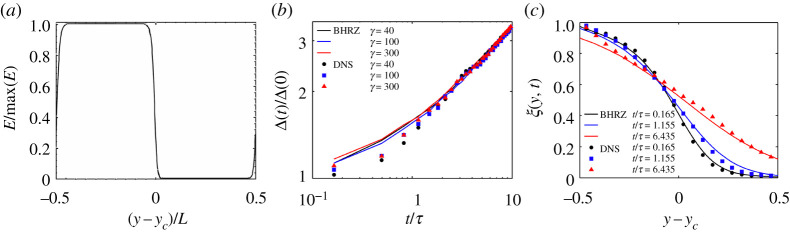
Initial condition specification for the SFML and some quantitative comparisons of DNS data [85] with the model. τ is the integral time scale of the initial field. (*a*) Initial spatial distribution of turbulent kinetic energy, E(y,t=0), in a SFML. (*b*) SFML mix-width for γ=300 (large initial inhomogeneity). (*c*) SFML energy for γ=300. Reproduced from [71]. (Online version in colour.)

Figure 2*a* illustrates the initial distribution of turbulent kinetic energy (TKE), E(y,t=0), in the SFML. The function satisfies E(y,t)=E(y+L,t), where y∈[0,L] and L is the periodic length scale. At t=0, the ratio of the maximum to the minimum TKE in the SFML is given by γ≡max[E(y,0)]/min[E(y,0)], and by varying γ, the strength of the initial inhomogeneity in the SFML can be controlled, and its effect upon the resulting flow can be examined. Note that in [85], only the TKE is inhomogeneous in the initial flow field; the integral length scale itself is homogeneous. For convenience a normalized energy function is defined

4.1 $$E(y,t)\equiv \frac{\text{min}[E(y,t)]-E(y,t)}{\text{min}[E(y,t)]-\text{max}[E(y,t)]},$$

such that E(y,t)∈[0,1], in terms of which a mixing-layer width may be defined at any given time,

4.2 $$\Delta (t)\equiv Y_{\left[1/4\right]}-Y_{\left[3/4\right]},$$

where $E(Y_{\left[1/4\right]},t)=1/4$, $E(Y_{\left[3/4\right]},t)=3/4$. The value of $C_{d}$ was chosen to optimize mix-width evolution from the LWN model when compared with the DNS data. In the study, it was found that the turbulence transport is by far the most dominant contribution to the evolution of Δ(t), even at the relatively low $Re_{\lambda }$ of the flow (≈45 at t=0). For the case with γ=1 (weakest initial inhomogeneity for which DNS data were available), the optimum value of $C_{d}=0.0145$. From figure 2*b,* it can be seen that this choice of $C_{d}$ gives good agreement between LWN and the DNS data at long times, both qualitatively and quantitatively. On physical grounds, it makes more sense to match the solutions at longer times since the turbulence inhomogeneity weakens with increasing time, and it is in the regime of weak inhomogeneity that the approximations invoked in LWN are most justified.

Figure 2*c* shows that the BHRZ model is also able to predict E(y,t) quite well, without explicitly tuning the diffusion coefficient any further. That the BHRZ model predicts the dependence of E(y,t) on y as well as it does is somewhat surprising given that the transport model is only strictly appropriate in the limit where the PDF of u(x,t) is weakly perturbed from a Gaussian, as per usual closure assumptions (that is, fourth-order correlations can be approximated by the product of second-order correlations); the results in [85] show that even for γ=40, the PDF of u(x,t) is quite far from being Gaussian. This suggests one of two things: either the contributions from higher-order cumulants of the field u(x,t) to the physical-space transport are small, or that the contributions from higher-order cumulants of the field u(x,t) to the physical-space transport generate a similar dependance on y across the mixing layer, such that their effect is subsumed in the value determined for $c_{D}$.

Overall, it was found that the model is able to capture the gross features of the SFML quite well for intermediate to long times, including the evolution of the mixing-layer width and turbulent kinetic energy. At short times, and for more sensitive statistics such as the generation of the velocity field anisotropy, the model is less accurate, as will be discussed further below.

## 5. Anisotropy

The LWN model by construction permits a partial consideration of a tensorially anisotropic scale-dependent statistical description of turbulence. Indeed, the second-rank tensor representation of the Reynolds-stress tensor in spectral space (inner product of two spin-1 vector spaces) alone has spin-0 (isotropic, namely the kinetic energy (trace)), spin-1 (anisotropic deviator on the diagonal) and spin-2 (anisotropic deviator on the off-diagnonal) irreducible representations in the SO(3) symmetry group. Here, and in the remainder of this section, we use the notation standardized in early use of the SO(3) symmetry group to represent tensor statistics in turbulence reviewed in [88,89]. However, the *a priori* averaging over angles in k-space eliminates the potentially infinite space of irreducible representations of continuous functions on the sphere; this limitation can be shown to have an impact on the comparisons with physical data.

The earliest application of LWN to anisotropic flows occurs in [69]. It was shown in that work the LWN model was able, with some effort to specify initial conditions, to recover in large part the experimental results of [90], in which homogeneous turbulence was subjected to successive plane-strain distortions in a wind-tunnel configuration. The model was shown to give quantitative agreement with experimental data for irrotational strains and qualitative agreement with data for homogeneous shear and freely decaying turbulence. For turbulence subjected to homogeneous mean shear, final asymptotic self-similar states were recovered that distributed the stresses anisotropically among the different spectral components according to the anisotropic shear imposed.

**Figure 3.**
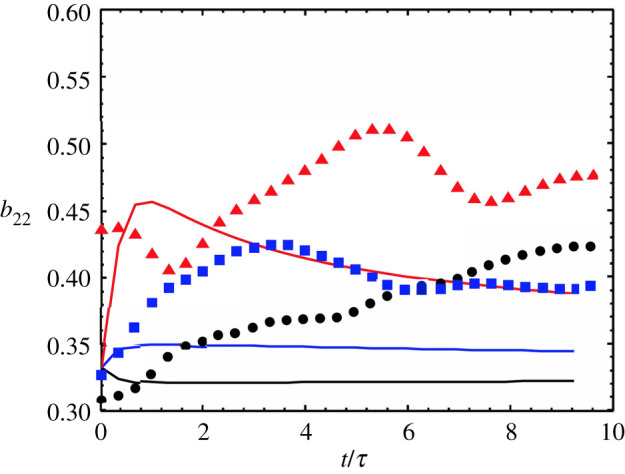
SFML anisotropy tensor component $b_{22}$ for DNS (symbols) and model (lines) at various positions in the mix-layer – $y-y_{c}=-2$ (black); $y-y_{c}=0$ (blue); $y-y_{c}=+2$ (red). Reproduced from [71] fig. 5. (Online version in colour.)

A more specific consideration of return-to-isotropy in the absence of mean-shear was done in the SFML comparison described above [71]. Using the value of $C_{d}$ optimized for mix-width evolution, the results for the anisotropy of the Reynolds stress $b_{ij}=R_{ij}/R_{nn}$ were measured for i=j=2 (the mixing direction). Figure 3 shows this quantity at different times (legend is the same as that for figure 2*c*). The main causes of the discrepancies are argued to be the angle averaging operations applied to the transport equations and the local approximation to the intrinsically non-local pressure-transport in physical space. Recent work [46,91] could provide a way to assess the effects of the angle averaging operation on the model prediction of the flow field anisotropy, and this work is underway and discussed further below. Incorporating non-local transport into the LWN model to overcome the deficiencies arising from the local approximation was explored in a later study [78], and it was shown that the effect was negligible. Therefore, it appears that anisotropy is the primary driver for the discrepancies observed here.

### (a) The rapid distortion limit and a local wavevector model

In recent work, the SO(3) group decomposition has been used to develop a computational scheme for the evolution of the general anisotropic two-point velocity correlation function in the linear problem of mean-flow coupled turbulence. This approach is distinct from previous uses of the SO(3) decomposition for turbulence in that the method is applied to the equations of motion to derive a computable model, instead of using the basis functions as a diagnostic tool in a post-processing step.

The pressure-strain correlations that arise in the (single-point) Reynolds stress evolution equations for incompressible flow are typically discussed in terms of a ‘rapid part’, that is, the terms that couple directly to the mean-flow gradients, and a ‘slow part’ that is expressed purely in terms of moments of velocity fluctuations [61]. Despite its linearity in the Reynolds stresses and in the mean velocity, the rapid pressure-strain (RPS) correlation represents a significant challenge to engineering turbulence modelling efforts due to its integro-differential nature; its representation in the single-point equations is unclosed and requires a model assumption to achieve closure. In the two-point description of the Reynolds stress evolution equation in k-space the RPS appears in closed form (see the fourth term in equation (2.11)), which can alleviate this issue. However, simplification of these two-point descriptions by describing the turbulence spectra as an average over all angles in Fourier space, so that the spectra are functions of wave number, rather than wavevectors, as in done in both LWN and EDQNM, results in the need to model the rapid part, despite its apparent simplicity.

Rapid distortion theory (RDT) is based on the assumption that, when a large mean-field distortion is imposed on a turbulent field, the early time response to the distortion can be described by ignoring the effects of higher-order velocity correlations. ‘Early time’ is defined in essence as the time while the turbulence time scales associated with the triple (and higher) correlations remain much larger than the time scales associated with the mean-field. Of relevance to this review is the derivation by Batchelor & Proudman [92] (BP54) of the exact form of the spectral correlation tensor in wavevector k-space as a function of the so-called extension factor, denoted here by c, a surrogate for the time over which the mean-strain acts on a homogeneous turbulence.

The potential disadvantage of the BP54 approach is that it is generally computationally costly. For the homogeneous case, it requires the solution of a fully three-dimensional wavevector problem. This difficulty is overcome to a degree by using the SO(3) decomposition in the approach described in [93], which reduces a three-dimensional problem of tensor functions of wave vector into a one-dimensional problem of computing scalar functions of the wavenumber. The code used to compute the results presented in [93] is built on the analysis and strategy developed in [94,95]. We will not review here the great deal of extra detail in those reports that are freely available to the interested reader. We will instead focus on the outcome of the computations and their implications.

**Figure 4.**
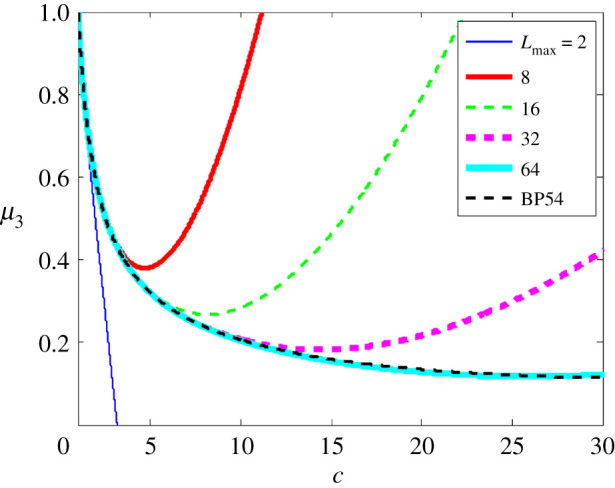
Ratio of post- to pre-distortion energy $\mu_{3}=R_{33}(t)/R_{33}(0)$ for various values of $L_{\text{max}}$ as a function of the distortion factor $c(t)=\exp\int_{0}^{t}(\partial U_{3}/\partial x_{3})dt$. BP54 is the theoretical result from [92]. c=exp≈2.718 corresponds to t=1, which is the initial large eddy turnover time. Reproduced from [93]. (Online version in colour.)

The computations reported in [93] were performed using the above strategy using a maximum of 64 rotational modes. Figure 4 shows that as the number of rotational modes in the calculation is increased, the agreement with BP54 endures for longer times and arbitrarily small error may be obtained with a finite number of rotational modes up to extension factor c=30 (which corresponded to roughly 11 large eddy turnover times, well beyond when RDT physics might be expected to hold in a realistic problem).

Calculation of the mean-flow coupled problem to arbitrary accuracy in the manner proposed in [93] points to strategies for improved modelling. As already noted, capturing anisotropy is critical in efforts to accurately model the RPS correlation. However, the anisotropy arises from coupling of the various terms in the problem via the linear operator. An important conclusion, therefore, is that the RPS correlation cannot be modelled in isolation from the other anisotropy-generating terms of the problem. On the other hand, the LWV calculation shows a way to systematically generate anisotropy at the second-order level of description and therefore suggests a suitable truncation at an order dictated by the problem itself. This observation, along with the understanding that the nonlinear terms, once included, would serve to ‘scramble’ the larger degrees (higher modes) of anisotropy, give rise to the possibility of a practical model with a small additional number of modes. One such is presented in [96] (MCS), in which an EDQNM model extension is proposed that includes the leading order anisotropy (spin-2) and the result is evaluated against homogeneous shear flow data, and successes and limitations are discussed.

From the point of view of the LWV computational model and code, these truncations are easily achieved by prescribing $L_{\text{max}}=2$ or 4 and such strategies are being currently pursued by the present authors. An obvious strategy to extend the LWN model to accomodate leading order anisotropy generation is to use something like the MCS strategy, which, because of the absence of nonlinear effects, local or otherwise, would be identical in the LWN framework. Such efforts are currently underway.

### (b) Cascade transfer term: an anisotropic Leith model for homogeneous turbulence

The models invoked for cascade models have either been restricted to isotropic statistics, as in the energy transfer models described in [97], or have treated anisotropy in a rudimentary manner: for example, the LWN model as we have laid out in §2a simply adds anisotropic terms suggested by single-point models to the isotropic Leith diffusion model. Although the result is useful, it seems incomplete, because logically, single-point models should be deduced from two-point models, not the other way around. It therefore seems reasonable to investigate whether a diffusion model similar to the Leith model could apply to anisotropic turbulence.

The purpose of the work described in [91] is to generalize the Leith model to anisotropic statistics more systematically by carrying out the arguments of [98] without assuming isotropy. Their analysis was based on the directional-polarization decomposition of the correlation tensor due to Cambon & Rubinstein [43]: this is a generalization of the trace-deviator decomposition for solenoidal tensors. The approximate analysis of local interactions was carried out following [98] to derive diffusion models for the directional and polarization components of the correlation tensor. Then substitute a low-order model for these components using the SO(3) decomposition [99] as developed in [46]; in the terminology of [46] this is the ‘spin-two’ part of the correlation, which alone contributes to the integrated Reynolds stresses (though, as demonstrated in the previous section, higher modes do contribute the *evolution* of the stresses).

**Figure 5.**
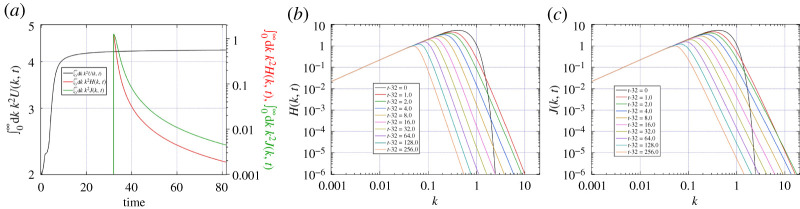
Quantification of decay of anisotropy using an extension of the Leith cascade mode to include the leading-order SO(3) representation terms in the evolution equations. (*a*) Time evolution of total energy (black) from t=0 and k-space integrated anisotropic components H(t) (red) and J(t) (green) initiated at t=32. Evolution of spin-2 (*b*) H(k) (directional anisotropy) and (*c*) J(k) (polarization anisotropy) starting from identical initialization at t=32. Reproduced from [91]. (Online version in colour.)

In this framework, the directional part of the spin-2 contribution to the Reynolds stress tensor is denoted by $H_{ij}$ and the polarization part by $J_{ij}$ and an equation was derived for each of these following the local interaction analysis strategy in [98]. In the test problem, the flow is driven until the energy goes to a constant. Then at time t=32 the forcing is stopped and the anisotropy is initialized identically for $H_{ij}(k)$ and $J_{ij}(k)$ and the anisotropy is allowed to decay according to the derived equations for each. This strategy was adopted so that the decay dynamics of H and of J could be isolated from the temporal dynamics of the energy. Figure 5*a* shows the decay of isotropy in such a test problem. They differ between the directional and polarizational components. Unlike a Rotta model [100], which would result in a linear damping of anisotropy in every wavenumber mode, figure 5*b* shows that the SO(3) decomposition in the spectral space allows for the anisotropy to populate initially isotropic modes as it cascades to the small scales before overall decay of the spectrum, during the return-to-isotropy process. This would not be possible in a Rotta-like model. Retaining the anisotropy and its cascade even in this low-order representation in spin-2 thus suggests the potential for more sophistication than linear damping in the return-to-isotropy mechanism.

## 6. Two-fluid mixing

We next turn our attention to recent efforts at validating the LWN model in variable-density two-fluid mixing applications. The mixing of fluids with different densities is an important process in many practical applications such as oceanic or atmospheric flows, combustion and inertial confinement fusion. Variable-density flows are those in which fluctuations of the density from its mean value are large and are two-way coupled with the velocity field. To predict the effects of such large density fluctuations on the mean flow and the turbulence in complex systems, we require high-fidelity computational models that are also economical to run [3,101,102]. Variable-density flows pose a difficulty beyond those encountered in incompressible single-fluid systems; the divergence-free condition of the velocity is lost and a pressure-strain correlation becomes non-trivial. Additional variables are introduced due to the coupling between the fluids and the driving mechanism. Variable-density flows have been studied in experiments [103–105], or using direct numerical simulations [106,107]. However, analytical models for such flows are mainly limited to single-point closure models [108–111], in which turbulence variables are studied as a function of a single space-point. The latter suffer the drawback of being incapable of capturing transients and scale generation [112], a fundamental feature of turbulence.

### (a) Homogeneous variable-density flows

In this section, we review our work in [81], which demonstrated the validity of the LWN model for a realistic problem and assessed whether and how accurately the modelling assumptions capture both integrated and spectral quantities. Since there are a total of eight modelling coefficients in the set-up for this problem (see §2b), they performed an optimization procedure that extracts a minimal set of four coefficients, which are sufficient to yield reasonable results while pointing to the mechanisms that dominate the mixing process in this problem.

To calibrate and test the spectral model, the authors used simulations performed with the CFDNS pseudo-spectral code [113]. The details of these simulations may be obtained in [114]. This flow represents a homogeneous version of the classical Rayleigh–Taylor instability and, during the growth stage, resembles the interior of the Rayleigh–Taylor mixing layer. The two fluids are initialized as random blobs, consistent with the homogeneity assumption. The initial density contrast is characterized by the Atwood number $At=(\rho_{1}-\rho_{2})/(\rho_{1}+\rho_{2})$, for heavy fluid of density $\rho_{1}$ and lighter fluid of density $\rho_{2}$, with the whole system driven by uniform acceleration (gravity). As the fluids mix due to molecular viscosity, the buoyancy forces decrease and at some point turbulence starts decaying. The non-stationary evolution of turbulence, resulting from the interplay between buoyancy turbulence production and mixing, is a challenge for one-point models [110].

In order to remain as systematic as possible given the relatively large number of tunable coefficients (see equations (2.20)–(2.23)), we first assign nominal values prescribed in [65,68]. The values of $C_{r1}=0.12$ and $C_{r2}=0.06$, their relationship constrained by [66], have the most prior validation due to studies of single-fluid homogeneous isotropic and anisotropic flows [68,69]. The corresponding spectral transfer coefficients for a ($C_{a1}$ and $C_{a2}$) and b ($C_{b1}$ and $C_{b2}$) were assigned to be identical to $C_{r1}$ and $C_{r2}$, respectively. The drag coefficients for $C_{rp1}$ and $C_{rp2}$ are set to unity in our work.

**Figure 6.**
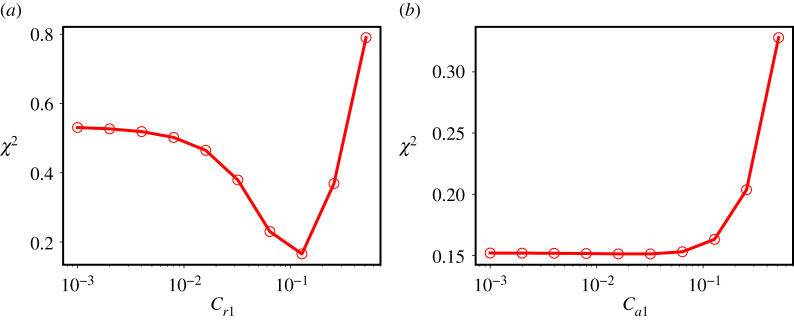
The Pearson’s $\chi^{2}$ function for different values of (*a*) $C_{r1}$ and (*b*) $C_{a1}$. The optimum value of each variable occurs at the minimum of the $\chi^{2}$ function. Reproduced from [81]. (Online version in colour.)

Each coefficient is optimized keeping the rest fixed and the quality of the optimizations for $C_{a1}$ and $C_{r1}$ are shown in figure 6*a*,*b*. There are two types of minima observed for the Pearson’s $\chi^{2}$ error function defined in [81]. The first is an asymptotic minimum as for $C_{b2}$, $C_{a1}$ and $C_{a2}$ (figure 6*a*) and the second is a true parabolic minimum as for $C_{rp2}$, $C_{b1}$ and $C_{r1}$ (figure 6*b*). Table 1 shows the optimized values (R1) of all coefficients for the At=0.05 case along with their uncertainties. For the cases that do not have a clear minimum the nominal values from [68] are retained and the range of uncertainty is taken to be all values between 0 and the first instance of the minimum. The values coefficients obtained by minimizing the error over all the dynamical variables simultaneously do not depart significantly from the nominal values proposed in [68]. Indeed, $C_{r1}$ and $C_{r2}$ (analogous to $c_{1}$ and $c_{2}$ in the single-fluid equations) are minimized at the theoretically expected values for constant density turbulence, which is a strong validation of the model and, less directly, of the assumption that the energy cascade in the variable-density mixing problem is not inconsistent with Kolmogorov dynamics.

**Table 1.** Table showing optimized values of all coefficients used for comparison with the DNS flow At=0.05 and At=0.75. Those values without uncertainties quoted correspond to $\chi^{2}$ minima that asymptotically approach zero.

parameter	$C_{b1}$	$C_{b2}$	$C_{a1}$	$C_{a2}$	$C_{r1}$	$C_{r2}$	$C_{rp1}$	$C_{rp2}$
R1	0.18±0.06	0.05	0.12	0.06	0.12±0.06	0.06	1.0	1.0±0.5

It appears that several of the eight coefficients, and hence the processes corresponding to the terms that they multiply, become subdominant, because of the manner in which they have asymptotically minimized errors for small values. The coefficients with true minima are $C_{b1}$, the downscale spectral transfer coefficient of b, $C_{r1}$ and $C_{r2}$, both of which govern upscale and downscale spectral redistribution of turbulence kinetic energy, and $C_{rp2}$, which provides a mechanism for the break-up of fluid parcels in scale due to turbulence. Using the coefficients obtained by optimization at At=0.05, we proceeded to compute the case of At=0.75 and found that the quantitative agreement with DNS is fairly good, similar to the low At case. Thus the optimization at low At also holds at high At, which is consistent with the fact that density contract (Atwood number) dependence is not explicit in the LWN model.

The emergence of four dominant coefficients leads to the understanding that, apart from the drive terms and the dissipation, which are treated exactly, the model expressions for downscale transfer of b, the break-up of fluid blobs as they sink under gravity and couple with the turbulence, and the resulting redistribution of E in spectral space, are the main mechanisms at play in the homogeneous variable-density mixing problem. At the level of second-order two-point correlations therefore, the model points to and helps elucidate the dominant physical mechanisms at play.

**Figure 7.**
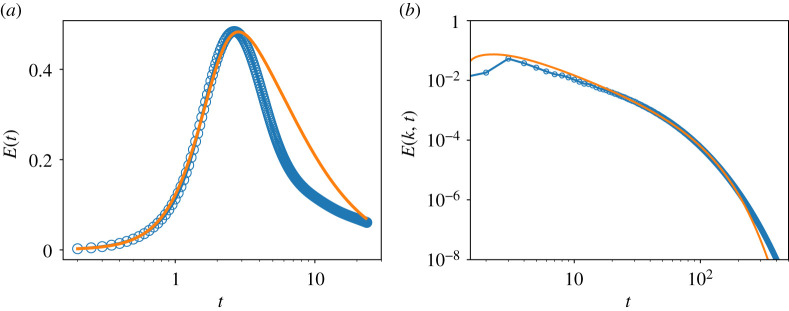
Comparison of LWN (orange line) against DNS data (blue symbols) for (*a*) time evolution of E(t) and (*b*) spectrum E(k) versus the wavenumber k at time t=3.0. (Online version in colour.)

The set of coefficients in table 1 obtained from analysis of the low At data appear to be suitable for a high Atwood number system At=0.75 as well. Figure 7 shows the mean turbulent kinetic energy computed by LWN compared with the DNS data for the same, showing both time-evolution in the mean and spectrum at an intermediate time of the flow. There is no approximation or assumption in the model development that requires Boussinesq or near-Boussinesq (low Atwood number) conditions. Therefore, it is perhaps not surprising that one set of coefficients works quite well over a broad range of At.

In summary, the LWN model is able to recover the statistical and spectral outcomes from non-trivial physical processes in a mixing problem, with minimal tuning of coefficients for two widely different Atwood number flows. The systematic study of the coefficients and a fairly simple optimization procedure has thus revealed useful constraints and properties both of the model as well as of the physical processes under study. These present a significant advantage in turbulence modelling.

### (b) Variable-density inhomogeneous mixing: Rayleigh–Taylor flow

**Figure 8.**
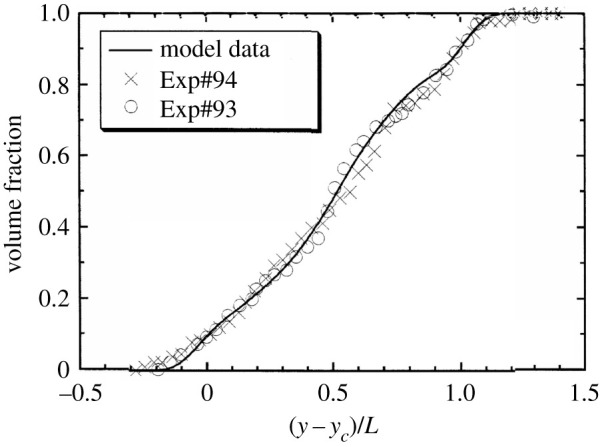
Volume fraction of the heavy fluid across the TMZ at a given time comparing the model (solid line) with experimental data from [115]. Reproduced from [77]

The most complex flow studied using the LWN model is that of Rayleigh–Taylor instability, an inhomogeneous variable-density mixing problem. The development of the LWN model for this case was completed in [65,77], resulting in equations (2.20)–(2.23). Figure 8 shows that the LWN model was able to recover the spatial distribution of the volume fraction of the heavy fluid in the experimental [115] turbulent mixing zone quite satisfactorily. There were no high-resolution data from simulations at the time. Recently, we have attempted a comparison of the model outcomes with high-resolution calculations of the Rayleigh–Taylor mixing layer with a particular focus on the effect of the source term in b (first term on the RHS in equation (2.23)) and the results are presented in [78]. It was shown that LWN performs very well at recovering the growth, peak and subsequent decay of b, as well as the mix-width, turbulence kinetic energy and mass flux velocity evolutions in time. These outcomes indicate that LWN holds significant promise as a practical model for instability-driven two-fluid turbulent mixing problems with wide applicability.

## 7. Conclusion

This review has surveyed the literature on the LWN spectral closure model, focusing particularly on the past 6 years of effort, which was nevertheless built on a nearly 30-year history of the model. We have attempted to show that over a period of time the model has been exercised via comparisons to a variety of experimental and, more recently, high-resolution DNS data. While there is not yet a universal or optimal set of coefficients that has been prescribed for the model, it does perform quite well for a remarkable number of flows, ranging in complexity from homogeneous and isotropic through inhomogeneous variable-density. In all cases, both the mean quantities as well as the spectra are recovered with physically reasonable values for the tuned coefficients (i.e. ≤O(1)) unless constrained by theory. It appears from the results of such efforts that this local spectral model might indeed be useful in computing leading order statistics in a range of practical applications.

The spectral nature of this model naturally invites a group-theoretic decomposition of the tensor variables as a function of wavevector. Recently, two potential approaches to compute anisotropy in the spectral model framework were developed: (1) a model to compute the mean-flow coupled anisotropic evolution (RDT limit) to arbitrary accuracy using rotational mode (SO(3)) decomposition and corresponding evolution of the tensor variables; and (2) an extension of the Leith model for anisotropic turbulence cascade, also using the tools of SO(3) group decomposition. These two models are logical enhancements of the LWN model and their integration into the modelling framework is pending.

Significant theoretical effort remains to be done in extending the anisotropic models for mean-flow and cascade to the case of variable density. Integrating inhomogeneity into the systematic description of anisotropic flow will also be a challenge for theoretical development. Sustained variable-density mixing needs the density-specific volume covariance b to be maintained to drive the mixing. Thus special care has to be taken to design the form of the source term in the b equation in systems where turbulence is sustained (such as Rayleigh–Taylor mixing). Model developments to incorporate the effects of material properties such as surface tension and immiscibility are also planned. Finally, extension of the model for compressible flows would be a leap forward but is particularly difficult in practice.

As the model continues to be developed and improved upon, we anticipate that the need for experimental and computation data for validation will become increasingly complex. In the near future, we plan to use existing high-resolution direct numerical simulation data [83,116] for both two-fluid KH and RM flows to validate LWN. Implicit large-eddy simulation data for KHI is also being generated using the MOBILE ILES code [117]. Numerical data are advantageous for LWN model validation because higher order quantities such as spectra can be extracted for more detailed comparisons. While experimental efforts do not carry the same granularity of information as simulations, the LANL also has invested resources in high energy density experiments to study shocked and sheared mixing [118], which could also be used as validation data for global mean quantities of interest. In the realm of single and two-fluid mixing, the data appear to be quite extensive. Extension to multi-species mixing would require a much more difficult class of experiments, which have yet to be executed.

As computational resources become more powerful, we aim to move past the limitations of the simpler single-point models to those such as LWN that are intrinsically more physical (in this case, carry scale information), but which also allow some flexibility and broad utility. The validation exercises performed with this model and reviewed above promise a robust framework for future applications and problems, as well as for studies focused on important mechanisms in turbulence and mixing.

## Data accessibility

The data presented in the paper have all been peer-reviewed elsewhere and are not new. We are happy to provide those data that were generated by us as needed but do not currently have a repository for these. The LWN code is not open-source at this time.
